# Nutrigenomic Functions of PPARs in Obesogenic Environments

**DOI:** 10.1155/2016/4794576

**Published:** 2016-11-30

**Authors:** Soonkyu Chung, Young Jun Kim, Soo Jin Yang, Yunkyoung Lee, Myoungsook Lee

**Affiliations:** ^1^Department of Nutrition and Health Sciences, University of Nebraska-Lincoln, Lincoln, NE, USA; ^2^Department of Food & Biotechnology, Korea University, Sejong, Republic of Korea; ^3^Department of Food and Nutrition, Seoul Women's University, Seoul, Republic of Korea; ^4^Department of Food Science and Nutrition, Jeju National University, Jeju, Republic of Korea; ^5^Department of Food and Nutrition and Research Institute of Obesity Science, Sungshin Women's University, Seoul, Republic of Korea

## Abstract

Peroxisome proliferator-activated receptors (PPARs) are ligand-activated transcription factors that mediate the effects of several nutrients or drugs through transcriptional regulation of their target genes in obesogenic environments. This review consists of three parts. First, we summarize current knowledge regarding the role of PPARs in governing the development of white and brown/beige adipocytes from uncommitted progenitor cells. Next, we discuss the interactions of dietary bioactive molecules, such as fatty acids and phytochemicals, with PPARs for the modulation of PPAR-dependent transcriptional activities and metabolic consequences. Lastly, the effects of PPAR polymorphism on obesity and metabolic outcomes are discussed. In this review, we aim to highlight the critical role of PPARs in the modulation of adiposity and subsequent metabolic adaptation in response to dietary challenges and genetic modifications. Understanding the changes in obesogenic environments as a consequence of PPARs/nutrient interactions may help expand the field of individualized nutrition to prevent obesity and obesity-associated metabolic comorbidities.

## 1. Introduction

In the past few decades, the prevalence of chronic diseases has been shown to be linked to nutrition deficiencies and overnutrition. Nutritional genomics/nutrigenomics, a unique approach for investigation of the genome-wide effects of nutrients at the molecular level, has contributed to the development of nutritional science and applications in medicinal and pharmacological research. Peroxisome proliferator-activated receptors (PPARs) are ligand-activated transcription factors (TFs) that mediate the effects of several nutrients or drugs through transcriptional regulation of their target genes. PPAR isotypes of the NR1 family, such as PPAR*α* (nuclear receptor; NR1C1), PPAR*β*/*δ* (NR1C2), and PPAR*γ* (NR1C3), can be distinguished based on their different biological roles and are the most relevant subtypes in the field of nutrition research. PPARs exert their biologically distinct functions in an isotype- and tissue-specific manner; however, the molecular details of tissue-dependent PPAR function remain unclear. PPARs are also able to repress transcription by interacting with other TFs and/or coactivators, thereby interfering with other signaling pathways to control physiology. Understanding the changes in the obesogenic environment as a consequence of PPAR/nutrient interactions may help expand the field of individualized nutrition to prevent obesity and its associated metabolic comorbidities.

In this review, we summarized current knowledge regarding (1) the role of PPARs in governing the development of white and brown/beige adipocytes from uncommitted progenitor cells, (2) interactions between dietary bioactive molecules and PPARs for the modulation of PPAR-dependent transcriptional activity and metabolic consequences, and (3) the effects of PPAR polymorphisms on obesity and metabolic outcomes.

## 2. Transcriptional Regulation of PPARs in White, Brown, and Beige Adipose Tissue

### 2.1. Functions of PPARs in White Adipose Tissue

#### 2.1.1. Regulation of Adipogenesis

The process of adipogenesis is divided into two distinct stages: determination and terminal differentiation. Each stage is governed by the orchestrated regulation of TFs. TFs involved in the stage of adipocyte determination include CCAAT/enhancer-binding protein *β* and *δ* (C/EBP*β* and C/EBP*δ*), glucocorticoid receptor (GR), signal transducer and activator of transcription 5A (STAT5A), and cAMP-response element-binding protein (CREB) [22, 23]. These TFs induce the transcriptional activation of target genes responsible for the second stage of adipogenesis. Regulators of early-stage adipogenesis, that is, C/EBP*β* and C/EBP*δ*, directly induce the expression of C/EBP*α* and PPAR*γ*, which transcriptionally activate their own expression and the expression of other adipogenesis-related genes, for example, PPAR*γ* coactivator 1 alpha (PGC-1*α*) and fatty acid synthase (FAS) [24–26].

PPAR*γ* is known for its role in the regulation of adipogenic and lipogenic pathways [4, 27, 28]. Initial studies examining the role of PPAR*γ* in adipogenesis showed that PPAR*γ*-knockout mice had little adipose tissue [29]. PPAR*γ* cooperatively acts with early adipogenic TFs, such as C/EBPs [30]. C/EBP*β* and C/EBP*δ* induce PPAR*γ* expression, and C/EBP*α* and PPAR*γ* commutatively induce the expression of each other by facilitating chromatin binding [4, 31]. Some studies have suggested that the involvement of PPARs in adipogenesis is limited to the effects of PPAR*γ* during later stages of adipogenesis and terminal differentiation of adipocytes. However, evidence has shown that PPAR*γ* also plays a role in the early stages of adipogenesis. A subset of adipocyte progenitors is present within the WAT perivascular region in which PPAR*γ* is expressed, suggesting that this protein may have a role in adipocyte self-renewal [32, 33]. The involvement of PPAR*γ* in adipogenesis is more evident at the later stages of adipogenesis in mature adipocytes. Because the ablation of PPAR*γ* is lethal, cell-specific knockout of PPAR*γ* has been utilized in mature mouse adipocytes by applying the tamoxifen-dependent Cre-ER (T2) recombination system. A few days after ablation of the PPAR*γ* gene, mature adipocytes and brown adipocytes died, and a subset of PPAR*γ*-positive cells appeared [34], suggesting the involvement of PPAR*γ* in maintaining mature adipocytes.

#### 2.1.2. PPARs and Adipokines

PPAR*γ* controls the expression of adipokines, including adiponectin, leptin, fibroblast growth factor 1 (FGF1), FGF21, resistin, and tumor necrosis factor-*α* (TNF-*α*). Adiponectin is a main adipokine that stimulates insulin sensitization by increasing glucose uptake and decreasing gluconeogenesis. Between the two adiponectin receptors (AdipoR1 and AdipoR2), AdipoR2 activates hepatic PPAR*α* [35]. Hepatic PPAR*α* activation by AdipoR2 decreases lipid accumulation and lipid peroxidation, contributing to improvements in hepatic steatosis and nonalcoholic steatohepatitis [35]. Adipose PPAR*α* and PPAR*γ* activation increases adipocyte uptake of glucose and free fatty acids and enhances insulin sensitivity by inducing the expression of AdipoR1 and AdipoR2 [36]. In contrast to adiponectin, PPAR*γ* indirectly suppresses adipose leptin expression by inhibiting the binding of C/EBP to the leptin promoter region [37].

FGF1 is known to be selectively induced in adipose tissues by consumption of a high-fat diet through PPAR*γ*, which acts on adipose tissue remodeling [38]. The phenotypes of FGF1-knockout mice depend on the conditions. FGF1-knockout mice do not show abnormal phenotypes under normal physiological conditions; however, the mice show disruption of fat expansion and subsequent development of diabetes in an obesogenic environment from high-fat feeding. FGF21 is expressed in the adipose tissue and liver and exerts tissue-specific effects. In adipose tissues, FGF21 increases energy expenditure and prevents weight gain in diet-induced obese and* ob/ob* mice [39]. Moreover, FGF21 also sensitizes cells to the effects of insulin by increasing adipose PPAR*γ* activity [40]. The PPAR*γ* agonist rosiglitazone induces FGF21 in the epididymal WAT of C57Bl/6 mice, and adipose FGF21 stimulates PPAR*γ*, affecting adipogenesis and insulin sensitization. In the livers of these mice, FGF21 is induced by the PPAR*α* agonist GW7647, and hepatic FGF21 acts as a hormone, regulating carbohydrate and lipid metabolism [40]. FGF21-knockout mice exhibited lipodystrophy with little adipose tissue owing to the low expression levels of PPAR*γ* and its target genes.

Resistin is another adipokine that is upregulated in patients with type 2 diabetes and inflammation-related diseases [41]. Transcriptional regulation of resistin is governed by cooperative regulation of PPAR*γ* and C/EBP in murine adipocytes, but not in human adipocytes. Additionally, PPAR*γ* reduces the expression of proinflammatory cytokines in adipose tissue by acting on nuclear factor-kappa B (NF-*κ*B) signaling. PPAR*γ* inhibits NF-*κ*B activity and induces its degradation by direct binding to NF-*κ*B [42], leading to downregulation of the proinflammatory cytokines TNF-*α*, interleukin-6 (IL-6), and plasminogen activator inhibitor-1. As a transrepressive effect of PPAR*γ* and NF-*κ*B, NF-*κ*B also inhibits PPAR*γ* transcriptional activity by acting on histone deacetylase 3 [43].

#### 2.1.3. Regulation of PPARs in Subcutaneous and Visceral WAT

Three subtypes of PPAR, that is, PPAR*α*, PPAR*β*/*δ*, and PPAR*γ*, have tissue-specific regulatory functions. The tissue-specificity of each PPAR isoform is due to differences in the tissue-specific expression and specific target genes of each PPAR isoform. The tissue distribution, target genes, and main functions of PPAR subtypes are summarized in Table 1. Among the three PPAR subtypes, PPAR*γ* is highly expressed in adipose tissues and stimulates glucose uptake and adipokine secretion [1]. Through its interactions with adipokines, PPAR*γ* is involved in adipogenesis, lipid metabolism, glucose homeostasis, adipose remodeling, and WAT browning in WAT [2]. PPAR*γ* agonists have been used as oral hypoglycemic agents by sensitizing cells to insulin action; however, the application of these agents is often limited because of their effects on increasing body fat content [3]. The PPAR*γ* agonist thiazolidinedione (TZD) selectively induces the differentiation of immature preadipocytes in subcutaneous fat. Newly differentiated adipocytes are small and exhibit increased insulin sensitivity without altering the total weight of the WAT [44]. Interestingly, the opposite is true of visceral fat pads; TZD treatment decreases the numbers of large adipocytes in visceral fat by increasing apoptosis in large and relatively insulin-resistant visceral adipocytes [45]. Fat deposition in subcutaneous adipose tissue is relatively beneficial compared with increased visceral fat contents in terms of the risk of metabolic syndrome and cardiovascular diseases (CVDs). Lipoprotein lipase (LPL) has been suggested to be involved in PPAR*γ*-dependent fat redistribution from visceral to subcutaneous tissues in PPAR*γ* agonist-treated experimental models. The mass and catalytic activities of LPL are increased in subcutaneous fat, but not in visceral fat depots, accompanied by alterations in factors involved in the regulation of LPL activity, fatty acid transport, and lipogenesis [46, 47]. Nonetheless, in addition to increased subcutaneous fat, other side effects, including adverse cardiac outcomes, have been reported in patients receiving rosiglitazone, resulting in withdrawal of rosiglitazone from the market. Novel approaches have been applied for the use of PPAR*γ* agonist as antidiabetic drugs, including development of new types of PPAR*γ* agonists, dual PPAR*α* and PPAR*γ* agonists, and combination with agents that can suppress adipocyte differentiation and fat accumulation. In particular, dual PPAR*α* and PPAR*γ* agonists have been accepted to be promising for the treatment of type 2 diabetes with dyslipidemia. Administration of lobeglitazone and saroglitazar was effective in lowering HbA1c and in improving glucose control and lipid profiles in subjects of type 2 diabetes [48–51]. It does not mean that these dual PPAR*α* and PPAR*γ* agonists have no side effects. For example, weight gain was still observed in lobeglitazone-treated diabetic subjects [50]. Collectively, existing evidence suggests that the usage of dual PPAR*α* and PPAR*γ* agonists was relatively well tolerated and acceptable considering the balance between efficacy and side effects.

### 2.2. Regulation of PPARs in BAT

#### 2.2.1. Cellular Origin of Brown and Beige Adipocytes

At least two metabolically distinct brown adipocytes are found in humans: “classical brown” and “beige” adipocytes [52–54]. Classical brown adipocytes possess molecular attributes similar to interscapular BAT (iBAT) of rodents based on constitutive uncoupling protein-1 (UCP1) expression, homogeneous multilocular morphology, and a myogenic origin (Myf5^+^) [55, 56]. Conversely, beige adipocytes are differentiated from nonmyogenic lineage progenitors (Myf5^−^) and possess low levels of UCP1 expression under unstimulated conditions. Although there is still some controversy regarding the cellular identity, anatomical location, and recruitment versus transdifferentiation of beige adipocytes [57, 58], the metabolic relevance of beige adipocytes in terms of energy expenditure [52, 59] has well been established in response to environmental stimuli, such as low temperature [60, 61], and to physical activity [62]. Because BAT activity is negatively associated with adiposity, insulin resistance (IR), and aging, therapeutics targeting BAT recruitment and activity have attracted attention as a potential novel treatment strategy. PPAR*γ* regulates the general differentiation program and metabolic function of brown adipocytes as well as white adipocytes. Given the metabolic relevance of BAT in metabolism, we will summarize the role of the PPARs in regulating brown and beige fat development in this section. We also propose that reduced PPAR*γ* activity may explain the compromised BAT activities in obesity and metabolic syndrome.

#### 2.2.2. Transcriptional Regulation of PPAR*γ* in Brown and Beige Adipocytes

PPAR*γ* is the single most important TF that governs white adipocyte differentiation. However, PPAR*γ* alone is insufficient to drive the entire brown adipogenic transcriptional program, and its transcriptional partner, PR domain-containing protein 16 (PRDM16), is also required. Nonetheless, PPAR*γ* activity is essential for the development of both classical brown and beige adipocytes. Using a brown preadipocyte cell line, researchers have shown that PPAR*γ* binding to the PPAR response element of UCP1 is required for the transcriptional activity of UCP1 [63]. Inhibition of PPAR*γ* activity using a dominant-negative mutant promotes the whitening of interscapular brown fat [64]. In addition, genome-wide binding analyses have demonstrated that PPAR*γ* binds to many other genes unique to brown adipocytes, suggesting that PPAR*γ* binding to PPRE response elements in brown target genes confers lineage specificity during brown fat differentiation [65, 66]. In addition to promoting classical brown adipocyte differentiation, PPAR*γ* has also been implicated in white-to-beige conversion. Lack of functional PPAR*γ* activity leads to defective beige fat recruitment, suggesting that beige fat development is dependent on PPAR*γ* function [64]. Conversely, synthetic PPAR*γ* ligands, particularly those in the TZD class, are potent regulators of mitochondrial biogenesis and cause significant increases in brown-specific phenotypes in white adipocytes, including UCP1 upregulation and uncoupled respiration [67–73]. In later studies, Ohno et al. showed that the white-to-beige conversion by PPAR*γ* agonism could be explained by stabilization of PRDM16 protein, the master transcriptional regulator of brown adipocytes [74]. Interestingly, energy expenditure is not increased in TZD-treated animals. This could be due to the observation that TZD-mediated systemic lipogenesis overshadows the improved mitochondrial function and suppresses *β*3-adrenergic receptor- (ADRB3-) mediated activation* in vivo* [75–77]. Another important mechanism through which PPAR*γ* agonism enhances BAT activity involves the activation of SIRT1, a Sir2 homolog and NAD-dependent deacetylase [78]. Moreover, activation of SIRT1 and deacetylation of PPAR*γ* by resveratrol increase the recruitment of PRDM16, resulting in implementation of the transcriptional cascade for brown signature genes [74]. In addition to PPAR*γ*, PPAR*α* plays a role in brown adipocyte formation. Because PPAR*α* is a primary regulator of mitochondrial *β*-oxidation, it is not surprising that PPAR*α* expression levels are higher in brown adipocytes than in white adipocytes. Although PPAR*α* expression is often regarded as a downstream brown marker gene, other studies have demonstrated that PPAR*α* functions simultaneously with PPAR*γ* to increase the brown-specific expression of PRDM16, PGC1*α*, and UCP1 [79, 80]. PPAR*β*/*δ* is ubiquitous, showing highest expression in the gut, but is now thought to be important in exercise-induced white-to-beige conversion and thermogenesis [81]. In conclusion, the plasticity of adipocytes in response to different environmental stimuli is likely regulated by the dynamic associations among TFs (e.g., PPARs and PRDM16) and their coregulators (e.g., PGC1*α* and SIRT1). These interactions between environmental and transcriptional regulators determine the lineage commitment of adipogenic progenitor cells, that is, white, brown, and beige adipocytes, and the metabolic fate of existing adipocytes, that is, browning or its reversal, whitening.

#### 2.2.3. Compromised Activities of PPARs in Obese and Metabolically Unhealthy BAT

Chronic activation of ADRB3 is a key signaling event enhancing BAT activity and/or mass. In healthy humans, at least three distinct metabolic responses occur concurrently in response to ADRB3 signaling: (1) an increase in BAT activity in preexisting classical brown adipocytes, (2) metabolic switch of some, if not all, existing white adipocytes to beige adipocytes in subcutaneous fat, and (3) new beige adipocyte formation from adipogenic progenitor cells [82, 83]. Emerging evidence has revealed that these metabolic adaptations of BAT are preceded by cellular remodeling of WAT via type 2 innate immune responses, that is, IL-4 and IL-13 secretion [84, 85], M2 macrophage polarization [86], and local catecholamine production from macrophages and eosinophils [86, 87]. Unfortunately, BAT activity in humans is inversely correlated with body fat mass [88, 89], age, and blood glucose levels [90, 91]. Compromised BAT activation in conditions of obesity and metabolic vulnerability is associated with impairment of immunological remodeling in WAT upon ADRB3 activation. Given the critical role of PPARs in BAT regulation, the molecular mechanisms through which these defective immune responses affect the transcriptional regulation of PPARs/PRDM16 and the recruitment of these proteins to brown-specific target genes need to be defined. One of the most plausible and reasonable mechanisms for defective BAT activation in obesity involves the inverse regulation of NF-*κ*B and PPAR*γ* transactivation [92–95]. Toll-like receptor 4- (TLR4-) mediated NF-*κ*B activation in obesity severely impairs cold-induced type 2 immune responses [85], downregulates PPAR*γ* and PPAR*α* expression, and markedly reduces beige fat development [96]. Similarly, Goto et al. showed that IL-1*β*, which causes systemic IR, strongly reduces PPAR*γ* expression and blocks BAT development upon cold exposure [97]. Hence, pharmaceutical or nutritional strategies to restore PPAR activities by NF-*κ*B suppression should be revisited as a new approach to reinstate type 2 immune responses and PPAR/PRDM16 recruitment for beige fat development.

## 3. Effects of Nutrition on the Modulation of PPARs

### 3.1. Fatty Acids (FAs) and Their Derivatives

Food components that act as ligands for PPARs can show multiple effects, including antidiabetic, antiadipogenic, and anti-inflammatory effects [98–100]. A wide range of PPAR agonists have been identified; synthetic PPAR agonists, such as fibrates and TZD, as well as natural PPAR ligands, such as dietary FAs and their derivatives, have been shown to bind to and activate PPARs [101–103]. Indeed, fibrates and TZD are already used to treat hyperlipidemia and diabetes mellitus, respectively. Ligand-activated PPARs play a critical role in regulating metabolic activities associated with lipid metabolism, glucose metabolism, and the inflammatory state [99]. As shown in various ligand-binding assays, PPARs generally prefer to bind to polyunsaturated FAs, whereas saturated FAs are poor PPAR ligands. Thus, the activity of PPARs can be modulated by FAs derived from the diet; however, the capacity of FAs to activate PPAR-dependent gene transcription varies according to the type of FA [101, 104]. A variety of FAs and their derivatives as PPAR ligands are shown in Table 2.

FAs are ubiquitous biological molecules that act as metabolic fuels and essential components of cellular functions [101]. Although FAs are essential biological components, elevated levels of circulating FAs are closely related to most common metabolic disorders, such as CVD, hyperlipidemia, obesity, and IR [105]. Not all fats are created equal; high consumption of foods enriched in saturated FAs has been shown to be associated with the development of common diseases, such as coronary artery disease, obesity, diabetes, and cancer, whereas consumption of a diet high in polyunsaturated FAs (PUFAs), such as fish oil, appears to have protective effects against atherosclerosis and heart disease [106, 107]. As FA sensors, PPARs should also be considered when evaluating the distinctly different physiological effects of different FAs owing to small structural variations in FAs and their derivatives [99, 108].

PUFAs are classified as* n*-3 and* n*-6 FAs that have opposing effects in the modulation of receptor signaling and gene expression;* n*-6 (i.e., arachidonic acid [AA])-derived eicosanoids are mostly proactive, whereas* n*-3 (i.e., eicosapentaenoic acid [EPA])-derived eicosanoids are inhibitory [109]. After the essential FAs linoleic acid (LA,* n*-6) and *α*-linolenic acid (LNA,* n*-3) are consumed, they are further metabolized by various desaturases and elongases to generate long-chain FAs including AA, EPA, and docosahexaenoic acid (DHA;* n*-3) [110]. AA, EPA, and DHA are then further metabolized by cyclooxygenases, lipoxygenases, and/or epoxygenases to various FA-derived eicosanoids, some of which are listed in Table 2 as PPAR ligands [111]. FAs bind directly to PPAR*α* at physiologically relevant levels and induce transcriptional activation. In fact, unsaturated FAs and PUFAs bind to PPAR*α* in the *μ*M range, which can be achieved by dietary intake [108]. With regard to activation potency, the* n*-3 FAs EPA and DHA are more potent as* in vivo* activators of PPAR*α* than* n*-6 FAs [112–114]. Moreover, various eicosanoids can activate PPAR*α* with a stronger affinity than their PUFA precursors [103, 115, 116]. Recent findings have shown that acylethanolamides (AEs), such as anandamide (AEA), palmitoylethanolamide (PEA), and oleoylethanolamide (OEA), which are biosynthesized in the gastrointestinal tract, also act as PPAR*α* activators [117]. PPAR*α* activation by OEA results in appetite suppression and lipolysis, whereas activation of PPAR*α* by PEA results in anti-inflammatory effects [118]. Known PPAR*β*/*δ* ligands are similar to those for PPAR*α* with much lower levels of activation [108]. PPAR*γ* ligands include unsaturated FAs, such as LA, LNA, CLA, DHA, and EPA, as well as FA derivatives in the physiologically relevant range [119]. Additionally, PPAR*γ* agonists can have systemic anti-inflammatory effects [100]. For example, the prevention of high-fat or high-energy diet-induced adipose tissue inflammation and remodeling by long-chain* n*-3 PUFAs is reported to involve PPAR*γ* activation [120, 121]. Collectively, various FAs and their derivatives are natural ligands for PPARs, with a fair amount of overlap among the three PPAR subtypes, and these molecules act as metabolic regulators by controlling the PPAR activity. Although many studies have helped to elucidate the role of PPARs in FA-mediated activation, more research is needed to determine the tissue distribution of PPAR subtypes in humans and evaluate the concentration and availability of FAs and their derivatives in human tissues.

### 3.2. Conjugated Linoleic Acids (CLAs)

CLAs are FAs that are mainly found in foods derived from ruminant animals [122]. CLAs are geometrical and positional isomers (*cis-* or* trans-*double bond positioning at 7, 9; 8, 10; 9, 11; 10, 12; or 11, 13) of the parent molecule LA (*cis*-9,* cis*-12-18:2,* n*-6). The* cis*-9,* trans*-11 (9*Z*, 11*E*-octadecenoic acid, C18:2) isomer, also known as rumenic acid, is generated through biohydrogenation of dietary LAs by ruminant microflora and is the most abundant natural CLA isomer (over 75–80% of total CLAs). Due to their multiple health benefits, CLAs are currently being used as dietary supplements for altering body composition in humans and livestock [123, 124]; however, little is known regarding the mechanisms of these beneficial properties of CLAs.

CLA isomers are ligands for PPAR*α*, PPAR*β*/*δ*, and PPAR*γ* [125, 126], exhibiting differences in health benefits and PPAR activation [124, 127]. For example, 9*Z*, 11*E*-CLA is a potent PPAR*α* ligand in the low nM range and exerts potent anticancer effects [125, 128]. In contrast, 10*E*, 12*Z*-CLA causes adipocyte delipidation, IR, and inflammation by acting as a PPAR*γ* antagonist [129]. In addition, a mixture of CLA isomers as well as 9*Z*, 11*Z*-CLA and 9*Z*, 11*E*-CLA isomers can significantly activate PPAR*β*/*δ* in preadipocytes [108]. Thus, there are important cellular mechanisms that are able to differentiate subtle structural changes in various CLA isomers to allow tissue- and species-specific responses [130, 131]. Taken together, these findings support that CLA affects the production of eicosanoids either directly or indirectly, enhances PPAR*γ* activation, attenuates the NF-*κ*B pathway, and directly decreases proinflammatory cytokines to have beneficial effects on inflammation, ultimately influencing metabolic syndrome-related conditions, including obesity, IR, and atherosclerosis [132]. Thus, CLAs can directly exert anti-inflammatory effects by regulating the expression of inflammatory mediators, potentially through NF-*κ*B-dependent and/or PPAR*γ*-dependent pathways [133, 134].

### 3.3. Flavonoids

Flavonoids are a class of polyphenolic compounds that are secondary plant products [135]. The structure of flavonoids is based on C6-C3-C6, which involves two aromatic rings (A and B) linked to a heterocyclic ring (C) containing one oxygen and three carbons. Flavonoids are classified as flavanols, flavones, flavonols, flavanones, isoflavones, and anthocyanidins according to structural differences in the C ring. Many studies have reported the functionalities of flavonoids. One of the main functionalities of flavonoids is their antioxidant effects, for example, metal chelating activity [136], reactive oxygen species (ROS) scavenging [137, 138], antioxidant enzyme activation [139], and *α*-tocopherol reduction [140], which collectively result in inhibition of ROS-mediated cellular aging [141], inhibition of mutations [142], anticancer effects [143], inhibition of LDL oxidation and CVDs [144–146], and reduction of ischemic damage [147]. Moreover, many studies have examined the antiobesity effects of flavonoids with regard to energy expenditure and lipid metabolism [148–151]. However, additional studies are needed because the antiobesity effects of flavonoids are still unclear. Thus, in this review, we discuss the effects of flavonoids on PPAR*γ*-mediated obesity based on the role of PPAR*γ* as a master regulator of adipogenesis. Abundant evidence has shown that PPAR*γ* influences the adipogenic transcriptional cascade as a master regulator of adipogenesis [26, 152]. PPAR*γ* is also involved in glucose and cholesterol metabolism. Regulation of PPAR*γ* activation is a primary focus in studies of the control of obesity and type 2 diabetes. TZD, a synthetic ligand for PPAR*γ* activation, is used in the treatment of type 2 diabetes. However, because TZD has major side effects, such as edema, weight gain, and heart failure, many researchers have attempted to identify natural PPAR*γ* activators [153–155]; indeed, identification of effective therapeutic modulators of PPAR*γ* without side effects or with reduced side effects has become a major research focus. Many studies have investigated the therapeutic effects of natural substances owing to the potential or practical negative effects of synthetic medications. Natural substances originating from plants and fruits are traditionally used for the treatment of various diseases. Additionally, the value of natural substances as sources for new drug discovery is increasing because natural substances can be used as a therapeutic strategy to avoid side effects of synthetic drugs [156]. Taken together, these findings highlight the role of natural substances in PPAR*γ*-mediated mechanisms. In this review, we discuss recent reports of the effects of flavonoids on PPAR activity based on an antiobesity perspective.

Recent findings have suggested that dietary flavonoids inhibit adipogenesis during differentiation of preadipocytes and prevent obesity by downregulation of PPAR*γ* expression. Catechin significantly suppresses body fat accumulation and downregulates PPAR*γ* in visceral WAT [5]. Quercetin also downregulates PPAR*γ* in WAT but does not alter the amount of body fat [18]. Notably, most evidence has been reported from* in vitro* studies rather than* in vivo *studies. Several flavonoids, including hesperetin [12], isoflavones [13], licochalcone A [15], luteolin [16], quercetin [19], and tangeritin [21], have been shown to have inhibitory effects on adipogenesis during differentiation of preadipocytes into adipocytes, accompanied by downregulation of PPAR*γ*. Activation of PPAR*γ* induces upregulation of various downstream target genes involved in lipogenesis and FA synthesis. Although it in unclear whether the inhibition of adipocyte differentiation occurs directly through PPAR*γ* activity, it is feasible that flavonoids may effectively inhibit the transcriptional activity of PPAR*γ* by inhibiting adipocyte differentiation via downregulation of PPAR*γ* [13].

Recently, numerous natural substances have been reported to potentially modulate PPAR*γ* activity as a source of PPAR*γ* ligands; the natural compounds involved in mediating these effects have been identified as flavonoids, lignans, and stilbenes [157]. In particular, the role of flavonoids in the regulation of PPAR*γ* activity has been extensively studied owing to the agonist potential of these molecules. Moreover, several studies have reported and highlighted the role of flavonoids as latent PPAR*γ* agonists against GW9662 or T0070907 (PPAR*γ* antagonists) [6–8]. The agonistic effects of flavonoids on PPAR*γ*-mediated obesity, however, vary according to the chemical characteristics of the flavonoids. Some flavonoids selectively modulate PPAR*γ* activity and suppress adipogenesis or obesity [5, 12, 13, 15, 16, 18, 19, 21]. In contrast, other flavonoids promote adipogenesis by activation of PPAR*γ* [6–11, 14, 17, 20], as shown in Table 3. Inhibition of the transcriptional activation of PPAR*γ* by flavonoids is closely related to suppression of adipogenesis [13, 158]. Accordingly, PPAR*γ* activity can be altered through various pathways, including posttranslational modification, ligand type, or ligand-binding domain. For example, inhibition of PPAR*γ* phosphorylation at serine 273 by PPAR*γ* ligands leads to antiobesity effects with fewer side effects because serine 273 phosphorylation prevents the transcription of antiobesity genes [156, 159]. Although flavonoids are known to show agonistic effects toward PPAR*γ*, the detailed molecular mechanisms of their antiobesity effects have not been fully elucidated. Taken together, these findings support the importance of identifying novel flavonoids that modulate PPAR*γ* activity through posttranslational modification, for example, through phosphorylation, in order to improve our understanding of the interactions of flavonoids with PPAR*γ*; such studies are expected to enhance the therapeutic potential of flavonoids. Furthermore, additional studies of the PPAR-dependent effects of flavonoids on tissue-specific events, for example, in the WAT and BAT, are needed. Based on the importance of the tissue-specific roles of PPAR, as demonstrated in this review, future studies may focus on tissue-specific PPAR regulation by flavonoids.

## 4. Nutrigenetics and PPAR*γ* Polymorphisms

### 4.1. PPAR*γ* Gene and Polymorphism

*PPARα* is located on chromosome 22q13.3 and spans 93.15 kb. Single nucleotide polymorphisms (SNPs) in* PPARα*, that is, L162V, V227A, and intron 7G>C, are associated with metabolic features, such as dyslipidemia, CVD, and type II diabetes [160]. In this review, we have focused on* PPARγ* SNPs associated with the obesogenic environment because of the limited availability of information regarding the clinical/biological effects of genetic variants in* PPARα* and* PPARβ/δ*.

The* PPARγ* gene, which encodes a TF belonging to the same family of NRs as steroid hormone receptors, is a master regulator of the relationships between nutrients (such as FAs), prostanoids, insulin-sensitizing agents, susceptibility to obesity, control of peptides released from adipocytes, and insulin sensitivity [161]. In macrophages, PPAR*γ* has been shown to regulate the suppression of inflammatory cytokine production and improvement of insulin sensitivity [162]. Alternative promoter regions within the* PPARγ* gene allow the formation of three* PPARγ* subtypes:* PPARγ1*,* PPARγ2*, and* PPARγ*3. Although* PPARγ1* mRNA has been identified in many tissues, including the heart, liver, skeletal muscle, and adipose tissue,* PPARγ2* mRNA is abundantly expressed in adipose tissue, whereas* PPARγ3* mRNA is expressed in macrophages, epithelial tissue, and adipose tissue [163]. The* PPARγ* gene extends over 100 kb and includes nine exons, which give rise to three different PPAR*γ* transcripts with differential promoter usage and differential splicing (*PPARγ1*,* PPARγ2*, and* PPARγ3*). The* PPARγ1* and* PPARγ2* expressed during the differentiation of 3T3-L1 into adipocytes are derived from two alternative transcripts which share six identical C-terminal exons. Although PPAR*γ* is well known for its role in adipogenesis, it also plays a crucial role in maintaining normal physiology, including insulin sensitization.

This role of PPAR is consistent with many human genetic studies of various single amino acid mutations, such as Pro12Ala, Pro115Gln, Cys114Arg, Cyc131Tyr, Cyc162Trp, Val290Met, Pro388Leu, Arg425Cyc, C1431T, and Pro467Leu, which are located in several domains [164]. Of these identified mutations in the* PPARγ* gene, a common polymorphism occurs in the* PPARγ2*-specific exon B. The Pro12Ala polymorphism rs1801282 (C34G) and the silent C1431T mutation (His449His, CAC478CAT) are frequently observed in* PPARγ2*. Many mutations in the* PPARγ* gene are associated with obesity and diabetes-related phenotypes [165]. For example, the Pro115Gln mutation is associated with obesity but not IR, and the mutations Val290Met and Pro467Leu are related to severe IR but not obesity [164]. The CCA-to-GCA missense mutation in codon 12 of exon B of the* PPARγ* gene encodes an NH2-terminal residue that defines the adipocyte-specific* PPARγ2* isoform [166]. Obesity is a multifactorial disorder involving the regulation of food intake and energy expenditure, and ethnicity-dependent-genetic factors play significant pathogenic roles.* PPARγ* genes independently or dependently regulate the transcription of target genes involved in obesity-related processes, such as adipogenesis, IR, angiogenesis, and inflammation, in a tissue-dependent manner. Therefore,* PPARγ2 *gene polymorphisms influence obesity in a complex manner, likely involving ethnicity-dependent variations in obesity-related phenotypes.

### 4.2. The Common Pro115Ala Polymorphism in PPAR*γ* and Obesity

The Pro12Ala polymorphism in PPAR*γ2* was first identified in 1997, and a point mutation found in the B exon of the NH2-terminal of PPAR*γ* at position 12 (rs1801282) was shown to cause a moderate decrease in the transcription activity and adipogenic potential of this protein [162]. The rare allele frequencies are high in Caucasians (12%) and relatively low in Asians (4% of Japanese and 1% of Chinese) and African Americans (3%) [167]. The Ala allele generated by the Pro12Ala polymorphism is associated with obesity and confers a 25% reduction in the risk of type II diabetes, IR, and CVD in Caucasians [167]. However, although PPAR*γ* is associated with IR and type II diabetes, the 12Ala allele does not reduce the risk of diabetes in South Asians, Chinese, and Malaysians [168, 169]. The 12Pro-161T haplotype is associated with lower body mass index (BMI) and lower fasting serum triglycerides (TGs) in Koreans but not in Iranians [170, 171]. In a meta-analysis of BMI subgroups, the Ala allele was shown to be associated with an increase in 0.96 units for BMIs of 35 or more, and this association was observed in individuals with BMIs of 27 to less than 35 or with BMIs of 35 or more when the meta-analysis was restricted to Caucasians [172]; this pattern was not found in Asians. Further analysis suggested that this discrepancy may be explained by differences in body weight distributions and lifestyles of these ethnic groups [173]. In Italian population, carriers of the* PPARγ2* Ala allele were found to have higher BMIs and fat-mass levels than carriers of the wild-type allele, although a metabolically healthy profile was associated with the* PPARγ2* Ala allele due to the more favorable distribution of adipose tissue. Researchers also found that there was a genetic interaction between Pro12Ala and ACE I/D with regard to BMI and fat mass [174]. According to a gene-diet interaction analysis of the* PPARγ* Pro12Ala polymorphism, there is an inverse association between the PUFA to saturated FA (SFA) ratio (P : S) and BMI/insulin levels in Ala carriers. Because the mean P : S ratio varies by more than 10-fold, for example, from 0.11 in Hungary to 1.2 in Portugal, this ratio may be a more effective stimulator of adipogenesis in Pro carriers than in Ala carriers [175]. This study suggested that when the dietary P : S ratio is low, the BMI in Ala carriers is greater than that in Pro homozygotes. Moreover, although consumption of a PUFA-containing diet does not affect* PPARγ2* mRNA expression, individuals with the Pro12Pro genotype are more likely to benefit from consumption of a PUFA-containing diet [176]. Similarly, intake of monounsaturated FAs has been shown to have this effect in Ala12 allele carriers. A study in Québec, Canada, showed that total fat and saturated fat intake are positively correlated with body mass change in Pro12 homozygotes, whereas Ala12 allele carriers are protected from this change [177]. Moreover, the Ala12Ala genotype also associated with higher expression of* PPARγ2*,* LPIN1*, and sterol regulatory element-binding protein-1c mRNA compared with that in participants harboring the Pro12Pro genotype. Thus, it is possible that different dietary patterns between ethnic groups could modulate the relationship between BMI and this particular SNP.

Adiponectin, an adipocyte-derived hormone, is encoded by the adipocyte C1q and collagen domain-containing (*ACDC*) gene located in chromosome 3q27. Many studies have shown that adiponectin is reciprocally associated with central and peripheral fat distribution, IR, inflammation, and atherogenic lipid metabolites [178]. In a Danish study, several* ACDC* polymorphisms were found to be associated with body fat distribution, whereas Pro12Ala was found to be associated with body fat accumulation (overall adiposity). Additionally, the CC genotype of SNP-11377, an SNP in the promoter of the* ACDC *gene, was shown to interact with the homozygous Ala12Ala genotype to mediate BMI [179]. Cooperative interactions between the* ACDC *and* PPARγ* genes in the modulation of insulin sensitivity have also been demonstrated in a recent family-based association study, revealing significant interactions between SNP+45T/G of the* ACDC *gene and the Ala12 allele in a Taiwanese population; however, there was no evidence for this associated in the Italian population [180].

Among the SNPs rs10865710 (C-681G), rs7649970 (C-689T), and rs1801282 (C34G, Pro12Ala), the G allele of rs10865710 in the* PPARγ* gene is frequently observed and has been shown to be associated with increased susceptibility to nonalcoholic fatty liver disease (NAFLD) [181]. Despite ethnic differences in the prevalence of NAFLD, the incidence of NAFLD is known to primarily depend on lifestyle, dietary habits, and hepatic metabolic syndrome. Many genetic variations related to the obesogenic environment, including oxidative stress, inflammation, fibrogenic mediators, dyslipidemia, and IR, are involved in the pathogenesis of NAFLD [182]. The A12 allele is associated with lower fasting plasma glucose but does not affect blood pressure, BMI, or other metabolic parameters in Palestinian individuals. However, in obese patients, the 12Ala allele was associated with elevated total plasma cholesterol levels and a tendency toward increased low-density lipoprotein (LDL) cholesterol [183]. The* PPARγ* Pro12Ala polymorphism is associated with a reduced risk of myocardial infarction (MI) according to the Physician's Health Study but confers an increased risk of MI or cardiac death according to the Health Professionals Follow-Up Study [184]. Additionally, we found that the 12Ala variant of PPAR*γ*2 may influence CVD risk by affecting lipid metabolism in obese Palestinian individuals with type II diabetes [184]. Therefore, additional studies of the* PPARγ* Pro12Ala polymorphism are necessary to fully elucidate the role of* PPAR* genetics in obesity independent of CVD, particularly with regard to available pharmacological PPAR-targeted agents.

### 4.3. The Common C1431T Polymorphism in PPAR*γ* and Obesity

The C1431T polymorphism, also referred to as His447His, His447His, C161T, or CAC478CAT, is a silent mutation located in exon 6 of* PPARγ* and is considered a better predictor of fasting insulin levels and IR than Pro12Ala. The polymorphism C1431T has been shown to be associated with susceptibility to CVDs, diabetes, abnormal leptin concentrations, obesity, and metabolic syndrome and is associated with BMI [185, 186]. Although the C1431T polymorphism has not been extensively studied, the rare T allele has also been inconsistently linked to increases in weight. Because the Pro12Ala and C1431T polymorphisms are in linkage disequilibrium, both rare alleles are associated with increased body weight, and the overall effect is additive when these alleles occur together [187]. In Chinese patients with diabetes, the Pro12Ala and C1431T polymorphisms may not be major etiological factors for type 2 diabetes; however, the C1431T polymorphism is associated with overweightness or obesity, despite the observation that there are no differences in the frequencies of C1431T, Pro12Ala, and their haplotypes between patients with type 2 diabetes and control subjects [188]. Notably, the Ala12 allele is consistently associated with a lower BMI, whereas the T1431 allele is consistently associated with higher BMI in the Scottish nondiabetic population [186]. In contrast, the heterogenotype and Ala homogenotype of* PPARγ* Pro12Ala are significantly associated with higher risk of obesity, whereas the C1431T polymorphism is not significantly associated in individuals from northern India. None of the haplotypes are associated with morbid obesity [189]. In the Korean population, the Pro12Ala and C1431T SNPs have been shown to be associated with some parameters of metabolic syndrome in women [190]. In the EDEN mother-child cohort study, mothers homozygous for the T allele of C1431T were also more obese (24% versus 9%, resp.; *P* = 0.035), and three times more mothers had gestational diabetes (18% versus 6%, resp.; *P* = 0.044). Moreover, the Pro-T haplotype conferred the highest risk of gestational diabetes (odds ratio = 1.89, 95% confidence interval [CI] = 1.05–3.40), whereas the Ala-C haplotype was associated with the lowest risk of gestational diabetes (odds ratio = 0.12, 95% CI = 0.52–1.70) [191]. Additionally, one study showed that both the Pro12Ala and C1431T variants of PPAR*γ* are not associated with metabolic syndrome or obesity in a population from southern India [192]. However, in UK and Chinese individuals with coronary artery disease (CAD), the* PPARγ* C1431T polymorphism is significantly associated with CVD risk factors, such as fasting serum lipid profiles, in the context of variant genotypes (CT + TT) [193, 194]. Angiogram-positive patients carrying the T allele have significantly higher TGs, serum C-reactive protein, and fasting blood glucose levels, and obese patients harboring at least one CAT478 allele have higher leptin levels than other obese patients with similar BMIs, suggesting that the* PPARγ* gene may influence the levels of plasma leptin in obese individuals [195]. Finnish women with both Ala and 478CAT alleles have significantly more fat mass than women with other alleles. Thus, the CAC478CAT polymorphism is not associated with BMI or other variables related to obesity in different ethnic population. Previous studies on isoflavones have shown their potential antiobesity effects, although the mechanisms are not clear; accordingly, foods containing high levels of isoflavones, such as Korean fermented soy food (*Doenjang*), have been used as functional foods for the treatment or prevention of obesity in Korea [196]. In a clinical study of* Doenjang*, visceral fat area was significantly decreased by* Doenjang* supplementation in individuals with a mutant T allele of* PPARγ2 *compared with those harboring a C allele [197], suggesting that* Doenjang *interacted with mutant alleles of* PPARγ2* to exert antiobesity and antioxidative effects in obese individuals.

### 4.4. Rare PPAR*γ* Polymorphisms and Obesity

The Pro115Gln polymorphism, a very rare gain-of-function mutation in* PPARγ*, is associated with obesity but not IR. Because fibroblasts containing the Pro115Gln mutation accumulate 2.5 times more TGs than the corresponding wild-type cell line, we expected individuals with the Pro115Gln mutation would tend to be obese in field studies. A variant of rare Pro115Gln has been shown to be associated with increased BMI among obese individuals, an effect attributed to constitutive activation of the PPAR*γ* protein, which results in accelerated cell differentiation. Dominant-negative* PPARγ* mutations are associated with severe IR, hypertension, and alterations in lipid profiles (low high-density lipoprotein [HDL], high TGs) [198]. These studies implied that the Pro115Gln polymorphism has pathophysiological relevance in obesity; however, in the nationwide German Epidemiological Field Study, the Pro115Gln polymorphism was shown to have no relevant impact on morbid obesity [199]. The Val290Met and Pro467Leu polymorphisms are the best-characterized dominant-negative mutations of* PPARγ*2 and have been shown to dramatically reduce the transcriptional activity of* PPARγ*2* in vitro*, resulting in severe IR with increasing fat accumulation, hypertension, and reduced adiponectin levels [165].

Based on these interesting findings from previous studies, we plan to investigate the association of rare or unknown polymorphisms in the* PPARγ2* gene with BMI, obesity, and basal metabolic rates in obese individuals in the future.

## 5. Conclusion

The modulation of the PPAR activities poses significant impacts on metabolism, irrespective of the modifications that originated from external factors, such as hormones, temperature, excess nutrition, and PPAR-targeted drugs, or from genetic alternations such as polymorphism. Too much activation and too little activation of PPARs are both associated with improper fatty acid handling and maldistribution of fat, which leads to pathogenesis of metabolic diseases. In this review, we intend to provide an integrative view of PPAR regulation by summarizing the recent updates in PPAR regulation in white and brown fat, dietary ligands of PPARs and by incorporating common and rare PPAR polymorphism. We would like to emphasize that PPARs' unique function of depositing extra energy into white adipose tissue and burning out fats in brown/beige adipose tissue and muscle should be balanced for maintaining metabolic health. To reach this goal, wise and prudent usage of natural PPAR ligands through diet could be an option. Also, keen understanding in tissue- and subtype-specific regulation of PPARs is perquisite for the development of drugs to treat metabolic syndrome utilizing PPAR biology. With the advent of “omics era,” our knowledge in individual variation in metabolic susceptibility has been tremendously progressed. Therefore, the individual genetic modification of PPARs should be taken into consideration with their environmental modifiers for an innovative approach to prevent obesity such as precision or personalized medicine.

## Figures and Tables

**Table 1:** Tissue distribution, target genes, and main functions of PPAR subtypes.

PPAR isoform	PPAR*α*	PPAR*β*/*δ*	PPAR*γ*
Tissue distribution	Liver, heart, BAT	Many tissues (mainly in skeletal muscle, liver, heart)	PPAR*γ*1: many tissues PPAR*γ*2: WAT and BAT
Target genes	*ACAA2*, *ACAD*, *CPT1A*, *CPT2*, *ETFA*, *ETFDH*, *HADHA*, *HADHB*, *SLC25A20*, *SLC22A5*, *TXNIP*, *apoA-1*	*ACOX1*, *CPT1*, *LCAD*, *UCP1*, *VLCAD*, *CPT1*	*ACBP*, *ACS*, *aP2*, *CD36*, *C/EBPα*, *GLUT4*, *LPL*, *GyK*, *IRS-1*, *IRS-2*, *PEPCK*, *PI3K*, *STAT1*, *STAT5A*, *STAT5B*
Physiological functions	Fatty acid oxidation, amino acid catabolism, oxidative phosphorylation, lipoprotein synthesis [1, 2]	Fatty acid oxidation, oxidative phosphorylation, muscle type determination [3]	Adipogenesis, lipid metabolism, glucose homeostasis [2, 4]

*ACAA2*, acetyl-CoA acyltransferase 2; *ACAD*, acyl-coenzyme A dehydrogenase; *ACBP*, acyl-CoA-binding protein; *ACOX1*, acyl-coenzyme A oxidase 1; *ACS*, acyl-CoA synthetase; *aP2*, fatty acid binding protein 2; BAT, brown adipose tissue, *C/EBPα*, CCAAT/enhancer-binding protein *α*; *CD36*, cluster of differentiation 36; *CPT*, carnitine palmitoyl transferase; *ETFA*, electron transfer flavoprotein alpha subunit; *ETFDH*, electron transfer flavoprotein dehydrogenase; *GLUT4*, glucose transporter 4; *GyK*, glycerol kinase; *HADHA*, hydroxyacyl-CoA dehydrogenase, alpha subunit; *IRS*, insulin receptor substrate; *LCAD*, long-chain acyl-CoA dehydrogenase; *LPL*, lipoprotein lipase; *PEPCK*, phosphoenolpyruvate carboxykinase; *PI3K*, phosphoinositide 3-kinase; *SLC25A20*, solute carrier family 25 member 20; *STAT*, signal transducer and activator of transcription; *TXNIP*, thioredoxin-interacting protein; *UCP1*, uncoupling protein 1; *VLCAD*, very long-chain acyl-CoA dehydrogenase.

**Table 2:** FAs and their derivatives as PPARs ligands.

Receptor	PPAR*α*	PPAR*β*/*δ*	PPAR*γ*
Ligands	Saturated FAs (weaker)	Unsaturated FAs	Unsaturated FAs (LA, LNA, CLA, DHA, EPA)
	Unsaturated FAs (LA, LNA, PUFAs, including AA, EPA, phytanic acid)	Saturated FAs (much weaker) Prostacyclin	15-d-PGJ2 15-HETE
	Leukotriene B4	4-HNE	9-HODE
	8-HETE	4-HDDE	13-HODE
	8,9-Epoxyeicosatrienoic acids		
	11,12-Epoxyeicosatrienoic acids		
	OEA		
	PEA		

AA, arachidonic acid; CLA, conjugated linoleic acid; DHA, docosahexaenoic acid; EPA, eicosapentaenoic acid; 15-d-PGJ2, 15-deoxy-Δ12,14 prostaglandin J2; LA, linoleic acid; LNA, *α*-linolenic acid; OEA, oleoylethanolamide; PEA, palmitoylethanolamide; 4-HDDE, 4-hydroxydodeca-(2E,6Z)-dienal; 4-HNE, 4-hydroxy-2-nonenal; 15-HETE, 15(S)-hydroxyeicosatetraenoic acid; HODE, hydroxyoctadecadienoic acid.

**Table 3:** Summary of recent publications on the effects of flavonoids: adipogenesis and PPAR*γ* activity.

Flavonoid	Model	Effect	PPAR*γ* activity	Ref.
Catechin	Adipocyte differentiation in human bone marrow mesenchymal stem cells High-fat diet- (HFD-) induced obese SD rats	Adipogenesis ↑ Fat ↓	Activity ↑ Not measured	[5, 6]
Daidzein	3T3-L1 preadipocyte differentiation High-fat high-sucrose diet-induced obese C57BL/6J mice	Adipogenesis ↑ Adipocyte area ↓	Activity ↑ Not measured	[7]
Equol	3T3-L1 preadipocyte differentiation	Adipogenesis ↑	Activity ↑	[8]
EGCG	AML-I human preadipocyte differentiation	Adipogenesis ↑	Not measured	[9]
Fisetin	3T3-L1 preadipocyte differentiation	Adipogenesis ↑	Activity ↑	[10]
Flavanone	3T3-L1 preadipocyte differentiation	Adipogenesis ↑	Activity ↑	[11]
Hesperetin glucuronides	3T3-L1 preadipocyte differentiation	Adipogenesis ↓	Not changed	[12]
Isoflavonoids	3T3-L1 preadipocyte differentiation	Adipogenesis ↓	Activity ↓	[13]
Kaempferol	3T3-L1 preadipocyte differentiation	Adipogenesis ↓	Activity ↑	[14]
Licochalcone A	3T3-L1 preadipocyte differentiation HFD-induced obese ICR mice	Adipogenesis ↓ Body weight ↓ Plasma lipid ↓	Not measured Not measured	[15]
Luteolin	3T3-L1 preadipocyte differentiation	Adipogenesis ↓	Not changed	[16]
Pentamethylquercetin	3T3-L1 preadipocyte differentiation	Adipogenesis ↑	Not measured	[17]
Quercetin	HFD-induced obese Wistar rats	Plasma TG ↓	Not changed	[18]
Quercetin-3-*O*-(6′′-Feruloyl)-*β*-d-galactopyranoside	3T3-L1 preadipocyte differentiation	Adipogenesis ↓	Not measured	[19]
Sakuranetin	3T3-L1 preadipocyte differentiation	Adipogenesis ↑	Not changed	[20]
Tangeritin	3T3-L1 preadipocyte differentiation	Adipogenesis ↓	Not measured	[21]

TG, triglyceride; HFD, high-fat diet.

## Abbreviations

AA: Arachidonic acid
ACDC: Adipocyte C1q and collagen domain containing
ADRB3: *β*3-Adrenergic receptor
AE: Acylethanolamide
AEA: Anandamide
APC: Adipogenic progenitor cell
BAT: Brown adipose tissue
BMI: Body mass index
C/EBP: CCAAT/enhancer-binding protein
CLA: Conjugated linoleic acid
CREB: cAMP-response element-binding protein
CVD: Cardiovascular disease
DHA: Docosahexaenoic acid
EPA: Eicosapentaenoic acid
FA: Fatty acid
FAS: Fatty acid synthase
FGF: Fibroblast growth factor
GR: Glucocorticoid receptor
IR: Insulin resistance
IL: Interleukin
LA: Linoleic acid
LDL: Low-density lipoprotein
LNA: Linolenic acid
LPL: Lipoprotein lipase
MI: Myocardial infarction
NAD: Nicotinamide adenine dinucleotide
NAFLD: Nonalcoholic fatty liver disease
NF-*κ*B: Nuclear factor-kappa B
NR: Nuclear receptor
OEA: Oleoylethanolamide
PEA: Palmitoylethanolamide
PGC-1*α*: PPAR*γ* coactivator-1 alpha
PPAR: Peroxisome proliferator-activated receptor
PRDM16: PR domain-containing protein 16
PUFA: Polyunsaturated fatty acids
ROS: Reactive oxygen species
SFA: Saturated fatty acid
SIRT: Sir2 homolog
SNP: Single nucleotide polymorphism
STAT5A: Signal transducer and activator of transcription 5A
TF: Transcription factor
TNF-*α*: Tumor necrosis factor-*α*
TZD: Thiazolidinedione
UCP1: Uncoupling protein-1
VFA: Visceral fat area
WAT: White adipose tissue.
